# Illness perception, coping, and quality of life in early-stage Mycosis fungoides^☆^^☆☆^

**DOI:** 10.1016/j.abd.2020.05.008

**Published:** 2020-11-16

**Authors:** Oz Segal, Naama Trumper, Felix Pavlotsky, Gil Goldzweig, Aviv Barzilai

**Affiliations:** a Departament of Dermatology, The Chaim Sheba Medical Center, Tel Hashomer, Ramat Gan, Israel; b School of Behavioral Science, The Academic College of Tel Aviv-Yaffo, Tel Aviv-Yaffo, Israel

**Keywords:** Mycosis fungoides, Psychosocial impact, Quality of life

## Abstract

**Background:**

Mycosis fungoides is the most common type of cutaneous T-cell lymphoma. Most early-stage mycosis fungoides cases follow an indolent course, hence considered by doctors a relatively easy condition. However, since mycosis fungoides bears the title of cancer, patients might perceive it differently.

**Objective:**

To investigate patients’ illness perception, and its relationships to quality of life, depression, anxiety, and coping among early-stage mycosis fungoides patients.

**Methods:**

A cross-sectional questionnaire-based study was conducted. Patients from a single tertiary medical center completed the Revised Illness Perception Questionnaire, the MF/SS-CTCL Quality of Life scale, the Hospital Anxiety and Depression Scale, and The Mental Adjustment to Cancer Scale.

**Results:**

Thirty patients (25 males, five females, mean age 51.60) with stage I mycosis fungoides were enrolled. Mycosis fungoides had a little impact on patients’ daily life, quality of life, and levels of depression and anxiety, and they generally coped well. Disease understanding was low and was negatively correlated with impairment to quality of life and depression. Patients felt that stress and worry were features of the disease’s etiology.

**Study limitations:**

A small sample of patients was included.

**Conclusion:**

Patients with early-stage mycosis fungoides adapt well to their disease. Psychological interventions should be aimed at improving patients coping style and enhancing illness understanding, in order to maintain high quality of life.

## Introduction

Mycosis fungoides (MF) is the most common type of cutaneous t-cell lymphoma (CTCL), accounting for almost 50% of all primary cutaneous lymphomas. It is a rare disease, with an incidence rate of 6.4 per million persons in the United States.^1^ In most early-stage cases MF has an indolent clinical course, and a good prognosis. Dermatologists who are familiar with the disease may think it is a relatively benign condition, especially at its early stages, and convey it as such to their patients. However, since MF bears the title of lymphoma, it is important to understand how patients perceive it, and how it affects their lives, as research in this area is lacking.^2^ Also, it must be kept in mind that even patients with early-stage MF are required to undergo frequent medical evaluation, and are given treatments which can burden their lives and affect their perceptions and psychological outcomes.^3^ Advanced-stage treatments may even be more demanding.^4^

The concept of illness perception is based on the commonsense model of self-regulation (CSM).^5^ According to the model, when people become ill they create subjective beliefs about their illness. Traditionally, these beliefs are grouped into five dimensions: (1) identity − the label placed on the disease and the symptoms associated with it; (2) causes − ideas about how one becomes ill; (3) consequences − expected sequelae of the disease; (4) timeline − expectations about the duration and course of the disease; and (5) controllability/cure − beliefs about the extent to which the disease is amenable to control.^6^

Illness perception is a major regulatory factor affecting patients’ emotional world and health-related behaviors. For example, rheumatoid arthritis patients’ illness perception was found to affect psychological functions like depression, even more than the disease’s severity.^7^ Among patients suffering from asthma, treatment controllability/cure beliefs greatly influence treatment adherence.^8^ This shows that illness perceptions hold practical implications regarding disease management.

Illness perception was studied among a small sample of patients with CTCL, indicating they perceive their illness as long lasting; have more faith in their medical treatment than in their own ability to treat their disease; and that they do not understand their disease.^2^ Unlike the case of alopecia, vitiligo, psoriasis, and atopic dermatitis, the effects of illness perception on anxiety and depression, coping, and quality of life (QoL) have yet to be addressed in patients with MF.^9^, ^10^, ^11^ Paying attention to QoL of dermatology patients is of great importance since dealing with a skin disease, let alone a lymphoma, can be very difficult, and is considered the fourth leading cause of non-fatal disease burden.^12^

Coping strategies are the way patients alleviate stress when confronted with traumatic events such as being diagnosed and having to live with an illness. QoL is affected not only by the objective medical condition and the patients’ perceptions, but also by their coping style.^13^ Coping is also associated with levels of emotional distress.^14^

The present study aimed to investigate psycho-dermatology aspects of patients with early-stage MF. The primary goal was to assess illness perception, coping, anxiety and depression. Focusing on patients’ QoL, the secondary goal was to learn how these factors influence it.

## Materials and methods

This was a questionnaire-based study. The study was approved by the institutional review board (3925-17-SMC).

### Participants

Patients with early stage MF (stage I), diagnosed, treated, and followed-up at the medical center, who were informed about their disease at least four weeks prior to enrollment were recruited either at their visit to the outpatient or phototherapy clinics. Patients were debriefed and completed the questionnaires described below.

### Measures

To assess patients’ perception about their illness the authors used the Revised Illness Perception Questionnaire (IPQ-R).^6^ This scale is divided into three sections: identity, perspective, and etiology. In the identity section patients report symptoms they think are associated with their MF. The sum of these is the total for this section. In agreement with the scale’s instructions, symptoms relevant to MF were added. The perspective section evaluates patients’ perspectives across seven subscales: timeline-acute/chronic, timeline-cyclical, consequences, personal control, treatment control, illness coherence, and emotional representations. These subscales investigate patients’ views regarding the chronicity of MF, its course, the impact it has on their life, whether MF is curable by the patient and the treatment, understanding, and negative feelings the illness creates, respectively. Patients indicated their level of agreement with the statements on a five-point Likert scale (1 = strongly disagree, 5 = strongly agree), with high scores representing the dominancy of the subscale (Cronbach’s alpha = 0.43 − 0.88). For example, higher scores in acute/chronic subscale indicate that MF is perceived as a chronic condition. The last section consists of 18 items concerning the perceived etiology of the disease: psychological attributions (beliefs that the disease was caused by stress and worry), risk factors *e.g*., diet or environmental factors, immunity, and accident or chance. Patients also indicated their level of agreements with the statements on a similar Likert scale. Cronbach’s alpha was 0.06 for the latter and 0.59 − 0.92 for the rest, and therefore was omitted.

Coping was assessed using the Mini-Mental Adjustment to Cancer scale (MINI-MAC).^15^ This 29-item instrument consists of five subscales: hopeless-helpless, anxious preoccupation, fighting spirit, cognitive avoidance, and fatalism (Cronbach’s alpha = 0.62 − 0.88). The authors edited item 13 and deleted “I worry about the cancer resuming” for its irrelevancy. Patients were asked about their level of agreement with the statements at the moment of completing the questionnaire on a four-point Likert scale (1 = definitely does not apply to me, 4 = definitely applies to me).

QoL was assessed by the MF/SS CTCL-QoL scale.^16^ This 12-item instrument measures MF interference on health-related QoL. Patients were asked about the severity of their condition over the past four weeks on a five-point Likert scale (1 = not at all, 5 = very severe) (Cronbach’s alpha = 0.93).

Depression and anxiety were assessed by the Hospital Anxiety and Depression Scale (HADS).^17^ This 14-item instrument was designed to assess symptoms of anxiety and depression in non-somatic patients. Patients were asked about their level of agreement with the statements over the past week on a four-point Likert scale (1 = all the time, 4 = not at all) (Cronbach’s alpha = 0.64-.75).

### Data analysis

Statistical analysis was performed using SPSS v. 22. Pearson’s correlation coefficient was calculated in order to assess relations between continuous variables. A repeated measure (within subject) analysis with planned contrast was used to identify the most dominant perceived cause of the disease and the most employed coping strategy. A mediation model was explored, in which the relation between illness perception components (independent variables) and QoL (dependent variable) are mediated by coping mechanisms. The analysis was based on the procedure proposed by Hayes & Scharkow using a macro developed for SPSS.^18^, ^19^

## Results

A total of 30 patients (25 males, five females), all stage I MF, completed all questionnaires and were included in the study. One patient refused to cooperate. Demographics and clinical data are described in Table 1. The mean age was 51.6 (range 22 − 73) years, the median disease duration was 55 months, and median time from symptoms-onset to diagnosis was ten months. Most patients responded well only with phototherapy and topical corticosteroids (Table 1).

**Table 1:** Demographics and clinical data.

Variable	Mean (SD) or count (%), range
Age (in years)	51.60 (13.65)
Male	25
Female	5
Marital status	
Single	3 (10%)
Married	24 (80%)
Divorced	3 (10%)
Education (in years)	14.76 (3.04)
No. of children	2.17 (0.88)
Time since diagnosis (in months)	58.68 (64.73), 4 − 360
Time to diagnosis (in months)	20.28 (22.40), 1 − 84
Treatment response	75% complete response, 22% partial response, 3% no response
Treatment modalities^a^	
Topical corticosteroids	3.7% (100%)
Phototherapy	88.9% (96%)
Systemic retinoids (acitretin, bexarotene)	7.4% (18.5%)
Topical nitrogen mustard	7.4%

a At time of questionnaires completion (% as past treatment).

IPQ-R scores showed participants perceive MF as a chronic condition (3.7 ± 0.9) with a mild cyclical nature (2.84 ± 0.74) − rather realistic. Patients did not perceive MF consequences as major (2.78 ± 0.83) but did attribute a moderate emotional impact of their disease on their lives (3.1 ± 0.83). Although perceiving their personal control over the disease (2.86 ± 0.74) and their level of coherence (2.95 ± 0.86) as quite low, patients showed quite a strong belief in treatment control over the disease (3.59 ± 0.72). In the identity subscale (5.57 ± 3.11), symptoms most commonly reported were skin redness (93%), dry skin (86%), and itchiness and changes in skin color (70%). In order to investigate whether illness perception changes over time, newly diagnosed patients were compared to established patients (cut-off: two years). It was found that established patients perceived their illness as more chronic (t = -2.56, p < 0.05) in comparison to newly diagnosed patients. No other significant differences were found.

Regarding causal items, psychological attributions *e.g.*, stress and worry (2.68 ± 0.95) were significantly higher than risk factors (1.9 ± 0.47), immunity (2.24 ± 0.95), and chance (2.1 ± 0.82) (F_1,28_ = 10.08, p < 0.05). In addition, psychological attributions were positively correlated with anxiety and depression, consequences, and emotional representations.

Fighting spirit was the most common coping mechanism (3.34 ± 0.66, F_1,29_ = 50.36, p < 0.01) in comparison to avoidance (2.77 ± 0.95), fatalism (2.62 ± 0.92), anxious preoccupation (2.25 ± 0.83), and helplessness (1.4 ± 0.80).

Regarding QoL, 57.1% reported MF mildly interferers with their QoL, 16.7% reported a moderate interference, 9.5% reported a substantial or severe interference, and 9.5% reported MF to have no or low interference on their QoL.

Scores of the depression and anxiety subscales were 3.37 ± 0.4 and 2.85 ± 0.52, respectively, meaning patients were not depressed and only mildly anxious.

### Relationship between illness perception, psychological outcomes, and demographics

A positive correlation between duration of disease with MF being perceived as chronic, and a negative correlation with beliefs in treatment efficacy were found. The period of time elapsed from symptoms-onset to diagnosis and treatment modality did not correlate with illness perception components. Naturally, treatment response did correlate with illness perception; the better the response was, the less patients felt that MF affected their lives (r = 0.39, p < 0.05), and emotional burden was reduced (r = 0.40, p < 0.05). QoL negatively correlated with various illness perception subscales: identity, emotional representations, consequences, and psychological attributions and maladaptive coping (helplessness and anxious preoccupation). Depression and anxiety were also positively correlated with impairment to QoL. In contrast, QoL positively correlated with illness coherence.

Fighting spirit was found to be positively correlated with stronger beliefs in treatment control, and negatively correlated with emotional representations (experiencing negative feelings). Although not a dominant coping style, helplessness significantly correlated with most of the variables; it was positively correlated with anxiety and depression, identity, psychological attributions, emotional representations, and impairment to QoL, and was negatively correlated with illness coherence and beliefs in treatment control (Table 2).

**Table 2:** Significant correlations between all the study variables.

Variable	Psychological attributions	Risk factors	Fighting spirit	Helplessness	Anxious preoccupation	Avoidance	Depression	Anxiety	Quality of life impairment	Age	Time since Dx
Identity		0.37^a^		0.60^b^	0.44^a^		0.49^b^	0.48^b^	0.48^b^		
Timeline- acute/chronic											0.61^b^
Timeline- cyclical						0.42^a^					
Consequences	0.60^b^	0.41^a^		0.67^b^	0.63^b^		0.55^b^	0.73^b^	0.73^b^		
Personal control											
Treatment control			0.51^b^	-0.54^b^			-0.38^a^				-0.53^b^
Illness coherence	-0.42^a^		0.39^a^	-0.49^b^			-0.36^a^		-0.59^b^		
Emotional representations	0.57^b^	0.45^a^	-0.43^a^	0.65^b^	0.72^b^		0.60^b^	0.70^b^	0.57^b^	-0.43^a^	
Psychological attributions				0.60^b^	0.55^b^		0.44^a^	0.59^b^	0.67^b^		
Immunity				-0.39^a^					-0.44^a^		
Risk factors							0.38^a^	0.58^b^			
Fighting spirit											
Helplessness							0.83^b^	0.66^b^	0.79^b^		
Anxious preoccupation							0.75^b^	0.74^b^	0.70^b^		

a p < 0.05.

b p < 0.01.

**Figure 1:**
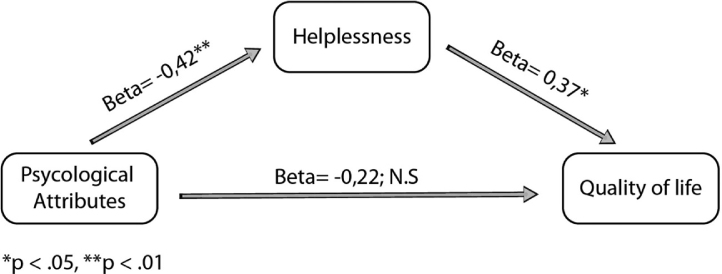
Coping style as a mediator between illness perception and quality of life.

The mediation model demonstrated that the significant effect of psychological attributions on QoL was mediated by helplessness. Controlling for helplessness results in non-significant relation between psychological attributions and QoL, *i.e*., the relation between psychological attributions and QoL was fully mediated by helplessness (Fig. 1).

## Discussion

Although MF can be a life-threatening condition, patients’ illness perception was optimistic. As opposed to patients with benign conditions, such as alopecias and psoriasis, here patients feel MF has less negative consequences, as was previously found.^20^, ^21^, ^22^ Unlike common (non-cancerous) dermatological patients,^22^ MF patients believe their treatment is effective – as was generally shown for patients with cancer, but unfortunately with time this feeling decreased. In addition to the positive picture, depression and anxiety levels were low, as opposed to the high prevalence of depression among dermatological patients, and QoL was high.^23^, ^24^, ^25^ Altogether, these findings may be partially attributed to the fact that in this study patients were stage I, which has an excellent response to skin-directed therapies and excellent prognosis.^22^ Possibly patients with advanced-stage MF would show a less positive profile.

Nevertheless, some findings should be addressed. First, most patient believe their disease was caused by stress and worry, similar to patients with atopic dermatitis.^26^ Attributing these psychological factors is in correlation with high levels of depression and anxiety, and to impairment in QoL. The mediation model shows that the negative effect that negative psychological factors have on QoL is mediated by the coping style of helplessness. It can be therefore assumed that the adverse effect of stress and worry on QoL can be eliminated by following adaptive coping styles such as fighting spirit. Furthermore, it seems that helpless patients believe that MF greatly impacts their lives and their emotional world. They have a poor belief in their treatment and in their self-ability to control MF, and their illness constitutes a fundamental component of their identity. The clinical implication of this is that enhancing adaptive coping styles are necessary in the management of patients.

Another disturbing finding that emerges is patients’ poor illness coherence. In contrast to patients with cancer, dermatological patients – including those in the current sample – do not feel they understand their diseases.^20^, ^21^, ^23^ One possible explanation is the relatively limited media and social discussions regarding MF, in comparison to cancer. When a condition is considered ambiguous, it generates more threat, which can cause patients to feel helpless and vulnerable. According to the CSM model, supplying patients with information regarding their illness and the necessary actions to deal with it are important for long-term adherence and better coping.^5^ In the present study it was found that illness coherence was negatively correlated with psychological attributions, helplessness, impairment of QoL, and depression. This means that there is a profound negative emotional and cognitive effect when patients feel that they do not properly understand their medical condition. Therefore, great importance should be placed on taking the time to educate patients about their illness.

One limitation of this study is that only patients with early-stage MF were included. It is unclear whether the conclusions can be generalized to patients with advanced-stage MF. However, the authors considered that it would be important to investigate early-stage patients, behaving like those with common skin conditions, rather than late-stage patients who might be more similar to cancer patients. Moreover, patients were recruited at the same clinic and were treated by the same expert. Doctors’ characteristics and communication style are known to have a strong effect on patients’ behavioral, emotional, and psychological outcomes, including satisfaction and QoL.^27^, ^28^ It is reasonable to assume that different doctors’ attitudes and messages result in different illness perceptions by their patients, especially when dealing with a rare disease. This advocates further investigations of the connection between doctors’ characteristics and patients’ illness perception.

## Conclusion

Generally, patients with early-stage MF hold favorable perspectives regarding their disease, cope well, and have low QoL-impairment. Nevertheless, patients who perceive negative psychological factors as an etiology of their disease and patients whose coping style is helplessness need suitable psychological interventions, as they might suffer from depression and impairment to their QoL. In the era of personalized medicine, focusing on individual mutations and biological-pathway therapies, patient’s psychological aspects should also be considered. More research should be done in order to expand the understanding of MF patients’ psychological needs, with an emphasis on illness coherence.

## Financial support

None declared.

## Authors’ contributions

Oz Segal: Approval of the final version of the manuscript; design and planning of the study; drafting and editing of the manuscript; collection, analysis, and interpretation of data; effective participation in research orientation; critical review of the literature; critical review of the manuscript.

Naama Trumper: Approval of the final version of the manuscript; design and planning of the study; drafting and editing of the manuscript; critical review of the literature.

Felix Pavlotsky: Approval of the final version of the manuscript; collection, analysis, and interpretation of data; effective participation in research orientation.

Gil Goldzweig: Statistical analysis; approval of the final version of the manuscript; collection, analysis, and interpretation of data; effective participation in research orientation; critical review of the literature; critical review of the manuscript.

Aviv Barzilai: Approval of the final version of the manuscript; design and planning of the study; drafting and editing of the manuscript; collection, analysis, and interpretation of data; effective participation in research orientation.

## Conflicts of interest

None declared.
